# Circulating Osteopontin Predicts Clinical and Radiological Response in First-Line Treatment of Advanced Non-Small Cell Lung Cancer

**DOI:** 10.1007/s00408-024-00675-5

**Published:** 2024-03-13

**Authors:** Davide Ramoni, Simona Coco, Giovanni Rossi, Chiara Dellepiane, Elisa Bennicelli, Sara Santamaria, Linda Zinoli, Alberto Stefano Tagliafico, Marco Tagliamento, Giulia Barletta, Luca Liberale, Amedeo Tirandi, Silvia Minetti, Maria Bertolotto, Fabrizio Montecucco, Carlo Genova, Federico Carbone

**Affiliations:** 1 First Clinic of Internal Medicine, Department of Internal Medicine, University of Genoa, 6 Viale Benedetto XV, 16132 Genoa, Italy; 2 U.O.S. Tumori Polmonari, IRCCS Ospedale Policlinico San Martino, 16132 Genoa, Italy; 3 IRCCS Ospedale Policlinico San Martino, U.O.C. Oncologia Medica 2, 16132 Genoa, Italy; 4 Dipartimento di Medicina, Chirurgia e Scienze Sperimentali, Università di Sassari, 07100 Sassari, Italy; 5 UOC Clinica di Oncologia Medica, IRCCS Ospedale Policlinico San Martino, 16132 Genoa, Italy; 6 Dipartimento di Radiodiagnostica, IRCCS-Ospedale Policlinico San Martino, 16132 Genoa, Italy; 7 Department of Health Sciences, University of Genoa, 16132 Genoa, Italy; 8 IRCCS Ospedale Policlinico San Martino, Genoa - Italian Cardiovascular Network, Genoa, Italy

**Keywords:** Lung cancer, Osteopontin, Immunotherapy, Pembrolizumab, Progression-free survival, Mortality

## Abstract

**Purpose:**

Pembrolizumab-based regimens are conditioned by the expression of PD-L1, but durable response rate is limited by innate and acquired resistance mechanisms. Here, we focus on osteopontin (OPN), an upfront biomarker of senescence, which closely associated with natural history of non-small cell lung cancer (NSCLC).

**Methods:**

Seventy-nine patients eligible to pembrolizumab regimens—alone or in combination with chemotherapy—as first-line treatment of advanced NSCLC were enrolled. Predictive value of OPN toward iRECIST progression disease (PD) was set as first outcome. Secondary ones included performance status (ECOG) at baseline, early (first and best) responses, and overall survival (OS).

**Results:**

High Serum OPN characterized patients with worse ECOG-PS (*p* = 0.015) at baseline and subjects experienced PD/death at first (OR 1.17 [1.02 to 1.35]; *p* = 0.030) and best responses (0.04 [0.00 to 0.81]; *p* = 0.035). OPN was associated with time-to-progression (B -2.74 [−4.46 to −1.01]) and time-to death (−0.13 [−0.20 to −0.05]). Cox regression models unveil a predictive value for iRECIST-PD (HR 1.01 [1.00 to 1.02]; *p *= −0.005), RECIST-PD (HR 1.01 [1.00 to 1.02]; *p* = 0.017), and OS (HR 1.02 [1.01 to 1.03]; *p* = 0.001). These models were internally validated through bootstrap resampling and characterized by relevant discrimination ability at ROC curve analyses.

**Conclusion:**

Baseline levels of serum OPN is closely associated with performance status and short/long term outcomes in patients with advanced NSCLC, which are candidate to pembrolizumab-based regimens. As upfront biomarker of senescence, OPN may pave the way for future studies focusing on senescence patterns in NSCLC.

**Supplementary Information:**

The online version contains supplementary material available at 10.1007/s00408-024-00675-5.

## Introduction

Therapy of non-small cell lung cancer (NSCLC) experienced significant breakthroughs in the last decade due to development of drugs targeting oncogenic drivers and progress in immunotherapy. Immunotherapy—alone or in combination with chemotherapy—is nowadays the gold standard treatment for NSCLC lacking a driver mutation [1]. The use of immune checkpoint inhibitors (ICIs) targeting programmed cell death protein 1 (PD-1) as single-agent or in combination with chemotherapy is conditioned by ligand (PD-L1) expression; indeed, in Europe the use of single-agent ICI (such as pembrolizumab, atezolizumab, cemiplimab) is reserved to patients whose NSCLC harbors high PD-L1 expression (≥ 50%), while combinations (such as chemotherapy plus pembrolizumab or chemotherapy plus ipilimumab plus nivolumab) are employed in case of PD-L1 between 0–49%. While ICIs are generally effective in this context, response may be lacking regardless of PD-L1 expression, and even patients achieving response or disease control are eventually expected to face disease progression sooner or later [2]. Resistance to ICIs may be due to innate or acquired mechanisms. Intrinsic inhibition to immune cell infiltration prevents antigen recognition by T-cell and patients do not respond to initial treatment. Late onset of acquired resistance accounts for relapse after an initial response. In both cases, tumor phenotype and microenvironment seem to have a role in providing a chronic immunosuppressive status that dramatically reduces response to treatment. Therefore, PD-L1 expression alone is not effective in predicting response, but other factors need to be considered: genomic drivers and mutational burden [3, 4], epigenetic and transcriptional signatures [5], T-cell infiltration and exhaustion [6, 7]. Identifying predictors of response/resistance is nowadays the greatest challenge in immune-oncology. A combination of tumor PD-L1 expression and mutational burden seems prognostic but not predictive of response [8]. Furthermore, they dynamically change, and immune-histological analyses are often limited by lack of tissue and standardized protocols. Rather, blood samples are non-invasive, quickly accessible, and easy to monitor over time. We here focus on osteopontin (OPN), a pleiotropic protein with established evidence in cancer biology [9], drug resistance [10], and clinical outcome [11]. We aim at establishing whether OPN displays a predictive value in terms of progression-free and overall survival (PFS and OS, respectively) in the first-line immunotherapy for NSCLC. Secondly, functional status at baseline and intermediate outcomes (i.e., first, and best responses) have been tested as surrogate index of resistance to treatment.

## Materials and Methods

### Study Design and Patient Selection

In this pilot study, patients in first-line treatment with pembrolizumab for advanced NSCLC, either alone or in combination with chemotherapy were consecutively enrolled at the Lung Cancer Unit of IRCCS Ospedale Policlinico San Martino, Genoa (Italy) between August 2017 and January 2021. In accordance with current guidelines [12, 13], all patients aged ≥ 18 years were enrolled if they had an established cyto-/histological diagnosis of NSCLC at advanced stage (IIIb and IV) and one or more lesions appraisable by classic RECIST (Response Evaluation Criteria in Solid Tumors) version 1.1 and/or immune-based RECIST (iRECIST) criteria [14]. Those with previous first-line therapy regimens, targetable oncogenic drivers (i.e., EGFR mutations or ALK/ROS1 rearrangements), steroids treatment (> 10 mg/day of prednisone or equivalent), and intrinsic contraindications to anti-PD-1 agents (e.g., organ transplantation, uncontrolled autoimmune disease) were excluded. Similarly, we did not include a control group of advanced NSCLC patients treated with no ICI. Patients eligible to chemotherapy alone are usually frail patients, introducing a potential bias in performance status and outcomes. Pembrolizumab was administered at a dose of 200 mg every 21 days, and was employed either alone or in combination with platinum-based chemotherapy, according to histology (cis/carboplatin plus pemetrexed for non-squamous histology or carboplatin plus paclitaxel/nab-paclitaxel for squamous histology) for up 4 cycles, after which patients with non-squamous histology received maintenance with pemetrexed plus pembrolizumab and patients with squamous histology received pembrolizumab alone for up to 24 months. Patients were followed-up over a median period of 10 months (range 0 to 49). Blood samples were collected before treatment starting and stored in accordance with GCLP guidelines [15]. Circulating levels of inflammatory biomarkers were measured on serum by ELISA, following manufacturer’s instructions (All DuoSet by R&D Systems, Minneapolis, MN). The lower limits of detection were 62.5 pg/mL for MMP-8, MPO, and OPN, and 31.25 pg/mL for MMP-9 and TIMP-1. The mean intra- and inter-assay coefficients of variation below 8%, as previously reported [16, 17]. The local Ethics Committee approved this protocol (PR180REG2017), performed in accordance with the guidelines of the Declaration of Helsinki. All patients gave an informed consent before enrollment.

### Endpoint Adjudication and Power Study Calculation

Primary endpoint of the study was testing the predictive value of serum OPN in first-line immunotherapy with pembrolizumab-based regimens: progressive disease (PD) was assessed by either RECIST and iRECIST criteria. At this regard, we acknowledge that our sample size (n = 79) does not satisfy the minimum required for developing a clinical prediction model so that our results should be considered as preliminary (Supplementary Material). Secondary outcomes included: i) a cross sectional association between OPN and performance status at enrollment as defined by the Eastern Cooperative Oncology Group (ECOG) performance status score of a 0 to 5-point scale (with higher scores indicating increasing disability); ii) perspective analyses of association between OPN and intermediate responses to treatment (1st and best response); iii) predictive value of OPN toward overall survival (OS).

### Statistical Analysis

IBM SPSS Statistics version 24.0 (IBM CO., Armonk, NY), GraphPad Prism version 9.0 (GraphPad Software, Inc, La Jolla, CA), and MedCalc for Windows version 12.5 (MedCalc Software, Ostend, Belgium) were used for statistical analysis. Clinical data were reported as absolute and relative frequencies when categorical, and comparisons drawn by Chi square or Fisher’s exact test. When the normality assumption – tested by Shapiro–Wilk – was not demonstrated, continuous clinical variables were presented as median and interquartile range [IQR] and intergroup comparisons drawn by Mann–Whitney and Kruskal–Wallis tests. Correlation analyses between continuous/ordinal variables were drawn by Spearman rank test, whereas linear regression models were built for extrapolating linear associations, reported as slope (B) and relative 95% confidence interval (CI). For outcome estimations we built univariate and adjusted regression models – logistic or multinomial, as appropriate – whose results were reported as odds ratio (OR) and 95% confidence interval (CI). Univariate and adjusted Cox proportional hazards models (expressed as hazard ratio [HR] and 95% CI) were finally built to test PD and OS. Discrimination properties of the models were then tested by ROC curve analyses presented as area under the curve (AUC), whereas internal validation with bootstrap resampling was used to avoid overfitting. A two-sided p-value < 0.05 was considered statistically significant for all the statistical analyses.

## Results

### Osteopontin is Closely Associated with Clinical Presentation of Advanced Non-Small Cell Lung Cancer

**Fig. 1:**
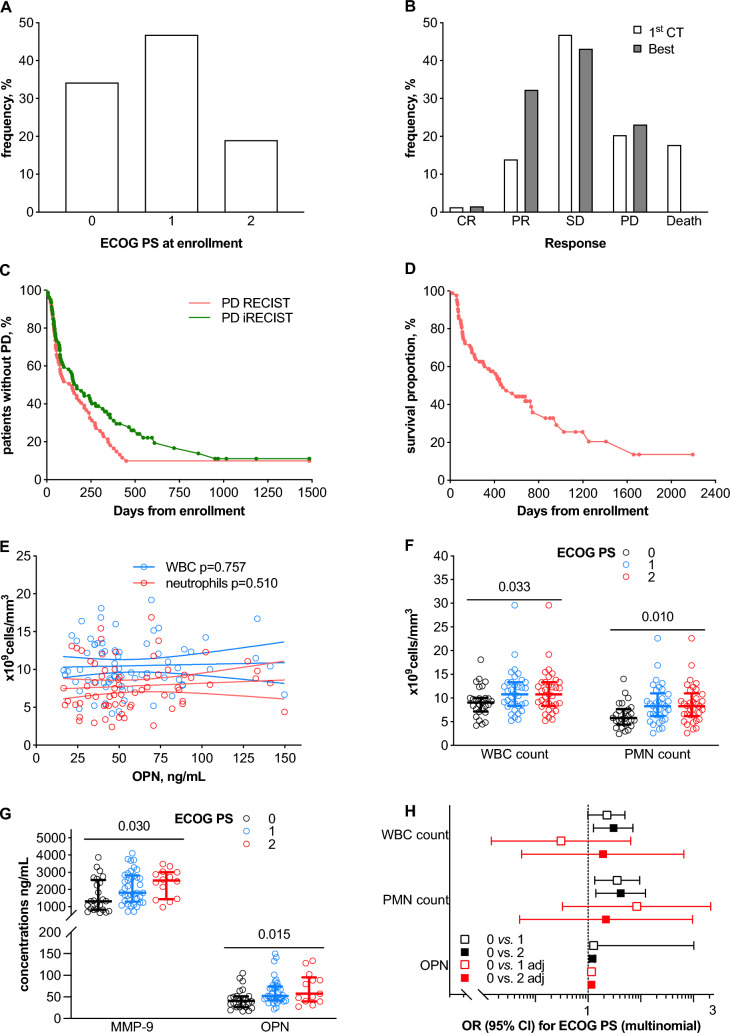
Osteopontin is closely associated with clinical presentation of advanced non-small cell lung cancer. Panels **A** and **B** summarized performance status according with performance status (ECOG; Eastern Cooperative Oncology Group) and early response defined by first/best responses at computerized tomography (CT) and categorized as: complete response (CR), partial response (PR), stable disease (SD), progression disease (PD) or death. Outcomes were classified according with immune-related criteria (iRECIST) (**C** and **D**). Association studies of osteopontin (OPN) with white blood cell (WBC) and neutrophil (PMN) counts, and ECOG as well are also reported (MMP: matrix metalloproteinase) (**E** to **H**)

Table 1 summarizes the characteristics of the study cohort. Patients received treatment with pembrolizumab alone (70.9%) or in combination with chemotherapy-based regimens. Median duration of treatment for pembrolizumab alone and combined therapy was 8 cycles [3 to 19] and 4 cycles [2 to 4], respectively. Subsequently, patients who received maintenance dual-therapy continued for a median of 5 cycles [4 to 13]. Performance status – according with ECOG scale – was < 2 in 81% of cases (34.2% and 46.8% for 0 and 1 score, respectively), whereas a score of 2 was recorded in 19.0% of patients (Fig. 1A). For about half of patients, stable disease (SD) was both the first and best responses (46.8 and 43.8, respectively), whereas partial response (PR) represented the best response for 32.3% of patients (Fig. 1B). By cut-off date, most of patients experienced PD with a rate of 86.1% and 78.5%, according to RECIST and iRECIST, respectively. Overall death incidence was instead of 65.8% (survival proportion are summarized in Fig. 1C and D). Concerning PD-L1 expression, no significant association with clinical parameters/inflammatory biomarkers were found, except for the different distribution across histotypes (squamous vs. non-squamous; *p *= 0.026) (Table S1).

**Table 1:** Clinical and histological ad radiological data of the study cohort (*n* = 79)

	Overall (*n* = 79)
Age, years [IQR]	71 [62–77]
Sex male, *n* (%)	59 (74.7)
Smoke	
never, *n* (%)	5 (6.3)
former, *n* (%)	38 (48.1)
active, *n* (%)	36 (45.6)
Histology	
SCC,* n* (%)	16 (20.3)
ADK, *n* (%)	63 (79.7)
PD-L1 expression	
None, *n* (%)	7 (8.9)
Mild, *n* (%)	14 (17.7)
High, *n* (%)	58 (73.4)
KRAS mutation, *n* (%)	28 (40.0)
Pembrolizumab single agent	56 (70.9)
Cycle number n [IQR]	8 [3–19]
Dual-therapy	23 (29.1)
Cycle number n [IQR]	4 [2–4]
Cycles maintenance, n [IQR]	5 [4–13]
WBC count, nx10E9/L [IQR]	9.9 [8.1–13.3]
Neutrophils, nx10E9/L [IQR]	7.5 [5.4–9.9]
Hb, g/dL [IQR]	13.2 [12.1–14.2]
Platelet, nx10E9/L [IQR]	311 [226–354]
CRP, mg/L [IQR]	21 [9–47]
OPN, ng/mL [IQR]	50 [38–74]
MPO, ng/mL [IQR]	697 [337–1211]
MMP-8, ng/mL [IQR]	56 [32–100]
MMP-9, ng/mL [IQR]	1814 [1197–2749]
Resistin, ng/mL [IQR]	18 [10–29]
TIMP-1, ng/mL [IQR]	314 [237–424]

Data are presented as median [interquartile range] or absolute (relative) frequencies

*ECOG PS* eastern cooperative oncology group performance status; *SCC* squamous cell carcinoma; *ADK* adenocarcinoma; *WBC* white blood cell; *Hb* hemoglobin; *CRP* C-reactive protein; *OPN* osteopontin; *MPO* myeloperoxidase; *MMP* metalloproteinase; *TIMP* tissue inhibitor of metalloproteinase

Correlation matrix (Figure S1) showed no significant relationship of OPN with neither white blood cell/neutrophil counts – also confirmed at linear regression analyses in Fig. 1 E – nor other inflammatory biomarkers. Nevertheless, both cell counts and markers of inflammatory response – but not other clinical variables – were generally associated with worse performance status: WBC (*p* for trend 0.033), neutrophils (*p* for trend 0.010), MMP-9 (*p* for trend 0.030), and OPN as well (*p* for trend 0.015) (Fig. 1F and G and Table S2). Cell counts and OPN also showed an independent association toward worse performance status at multinomial regression analysis with a slight – not significant – prevalence of OPN at adjusted analyses (OR 1.03 [1.00 to 1.06]; *p* = 0.066) (Fig. 1H).

### Baseline Values of Osteopontin Predict Short-Term Clinical Response to Therapies in Advanced Non-Small Cell Lung Cancer

**Fig. 2:**
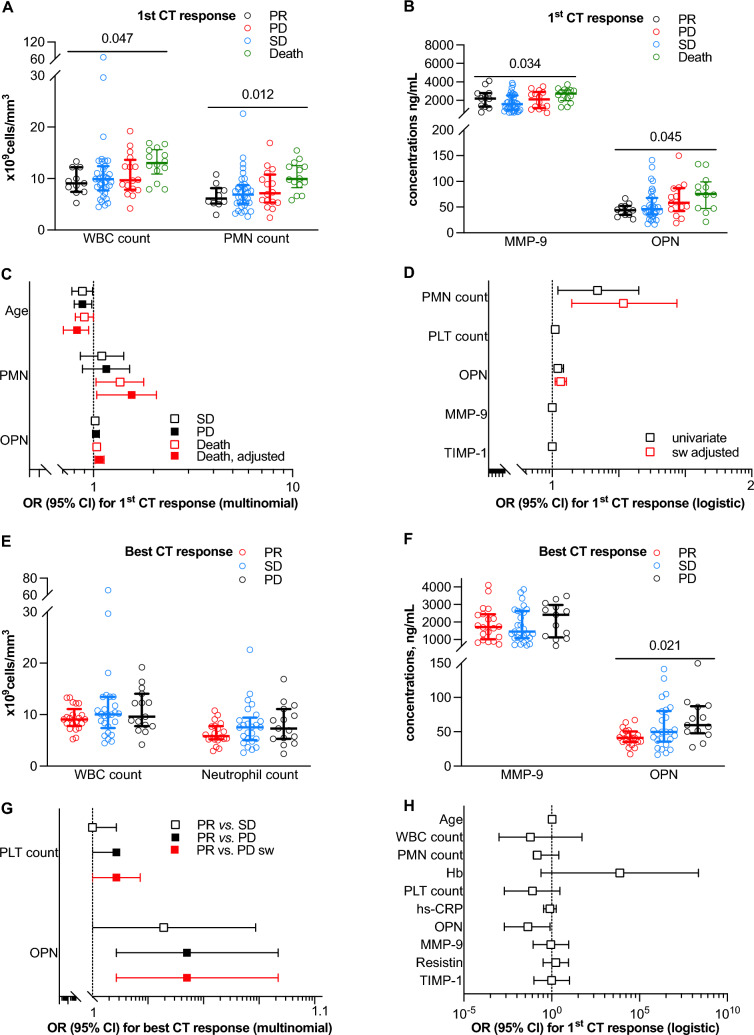
Osteopontin predicts early clinical response to therapies in advanced non-small cell lung cancer. White blood cell (WBC) and neutrophil (PMN) counts, matrix metalloproteinase (MMP) and osteopontin (OPN) were tested as predictor of and early response defined by first (**A** to **D**) and best (**E** to **H**) responses at computerized tomography (CT) and categorized as: complete response (CR), partial response (PR), stable disease (SD), progression disease (PD) or death. Outcomes were classified according with immune-related criteria (iRECIST) (**C** and **D**)

According to cross-sectional analyses, markers of inflammatory response were generally associated with first CT response (after excluding the only case of CR): WBC (*p* for trend 0.047), neutrophil count (*p* for trend 0.012), MMP-9 (*p* for trend 0.034), and OPN as well (*p* for trend 0.045) (Fig. 2A and B, and Table S3). At multinomial regression analysis, only age discriminated PR from both PD and early death with ORs of 0.88 (0.78 to 0.99; *p* = 0.029) and 0.90 (0.81 to 1.00; *p* = 0.043), respectively (Fig. 2C). A substantial discriminating value between PR and early death at first CT control was also observed for neutrophil count (OR 1.36 [1.03 to 1.79]; *p *= 0.031) and OPN (OR 1.04 [1.01 to 1.08]; *p *= 0.018), also at adjusted analysis (Fig. 2C). Once categorized (PR + CR vs. SD + PD), both neutrophil count and OPN remained independently associated with worse first CT response with ORs of 1.17 (1.02 to 1.35) and 1.02 (1.01 to 1.04), and *p*-values of 0.030 and 0.008, respectively (Fig. 1D).

When the best response was considered, only OPN increased progressively with the worsening of outcome (*p* for trend 0.021) (Fig. 2E and F, and Table S4) and discriminated PR to both SD (OR 1.03 [1.1 to 1.07]; *p* = 0.042) and PD (Fig. 2G). Even after categorization (PR + CR vs. SD + PD), only OPN retained a significant negative predictive value toward best response with a OR of 0.04 (0.00 to 0.81;* p* = 0.035) (Fig. 2H).

### Baseline Values of Osteopontin Predict Long-Term Clinical Response to Therapies in Advanced Non-Small Cell Lung Cancer

At Spearman correlation analyses, an inverse relationship with serum concentration of OPN at baseline was found for PFS (both RECIST and iRECIST) and OS (Table 2). Other relevant correlations involved performance status, WBC/neutrophil/platelet counts, and TIMP-1. Contrariwise, OPN was not associated with event rates (i.e., PD and death) as for performance status, cell counts, and neutrophil related degranulation products: MPO, MMP-8, and -9 (Table 3 and 4).

**Table 2:** Correlation of inflammatory parameters with time-to-progression (*n* = 68 and 62 for RECIST and iRECIST, respectively) and time to death (*n* = 52)

	PFS RECIST		PFS iRECIST		OS	
	**r**	**p-value**	**r**	**p-value**	**r**	**p-value**
Age	0.158	0.199	0.076	0.555	0.166	0.239
**ECOG PS**	**−0.252**	**0.038**	**−0.365**	**0.004**	−0.257	0.066
**WBC count**	**−0.246**	**0.046**	**−0.265**	**0.041**	**−0.276**	**0.050**
**Neutrophils**	**−0.337**	**0.006**	**−0.399**	**0.002**	**−0.430**	**0.002**
Hb	0.184	0.142	0.238	0.067	0.140	0.328
**Platelet**	−0.222	0.074	**−0.285**	**0.027**	− 0.270	0.056
CRP	−0.009	0.965	−0.076	0.723	−0.116	0.615
**OPN**	**−0.402**	**0.001**	**−0.417**	**0.002**	**−0.397**	**0.006**
MPO	−0.097	0.445	0.092	0.491	−0.087	0.549
MMP-8	−0.163	0.197	−0.091	0.498	−0.142	0.324
MMP-9	−0.217	0.085	−0.170	0.201	−0.237	0.098
Resistin	−0.055	0.665	0.087	0.518	0.141	0.328
**TIMP-1**	−0.231	0.066	**−0.266**	**0.044**	−0.214	0.135

Comparisons were drawn by Spearman correlation test

*ECOG PS* eastern cooperative oncology group performance status; *WBC* white blood cell; *Hb* hemoglobin; *CRP* C-reactive protein; *OPN* osteopontin; *MPO* myeloperoxidase; *MMP* metalloproteinase; *TIMP* tissue inhibitor of metalloproteinase

**Table 3:** Variables associated with RECIST/iRECIST response (*n* = 79)

	RECIST response (*n *= 11)	RECIST PD/death (*n *= 68)	iRECIST response (*n* = 17)	iRECIST PD/death (*n* = 62)	p-value*	p-value^§^
Age, years [IQR]	74 [65–79]	71 [62–76]	74 [67–79]	71 [62–76]	0.195	0.161
Sex, male (%)	8 (72.7)	51 (75.0)	12 (70.6)	47 (75.8)	1.000	0.755
Smoke			0.905		0.689	0.786
never, *n* (%)	1 (9.1)	4 (5.9)	1 (5.9)	4 (6.5)		
former, *n* (%)	4 (36.4)	34 (50.0)	7 (41.2)	31 (50.0)		
active, *n* (%)	6 (54.5)	30 (44.1)	9 (52.9)	27 (43.5)		
ECOG PS					0.200	0.295
0, *n* (%)	4 (36.4)	23 (33.8)	7 (41.2)	20 (32.3)		
1, *n* (%)	7 (63.6)	30 (44.1)	9 (52.9)	28 (45.2)		
2, *n* (%)	0 (0.0)	15 (22.1)	1 (5.9)	14 (22.6)		
Histology			0.774		0.446	0.500
SCC, *n* (%)	1 (9.1)	15 (22.1)	2 (11.8)	14 (22.6)		
ADK, *n* (%)	10 (90.9)	53 (77.9)	15 (88.2)	48 (77.4)		
PD-L1 expression						
None, *n *(%)	2 (18.2)	5 (7.4)	2 (11.8)	5 (8.1)	0.491	0.716
Mild, *n* (%)	2 (18.2)	12 (17.6)	2 (11.8)	12 (19.4)		
High, *n* (%)	7 (63.6)	51 (75.0)	13 (76.5)	45 (72.6)		
KRAS mutation, *n* (%)	4 (40.0)	24 (40.0)	7 (43.8)	21 (38.9)	1.000	0.776
Pembrolizumab single agent, *n *(%)	7 (63.6)	49 (72.1)	13 (76.5)	43 (69.4)	0.722	0.765
**WBC count, nx10E9/L [IQR]**	**8.6 [5.8–9.3]**	**10.5 [8.3 – 13.4]**	**8.6 [5.7 – 9.4]**	**10.9 [8.4 – 13.5]**	**0.007**	**0.003**
**Neutrophils, nx10E9/L [IQR]**	**6.0 [3.6–7.4]**	**7.8 [5.4 – 10.4]**	**5.5 [3.6 – 7.6]**	**8.0 – 5.8 – 10.8**	**0.030**	**0.009**
Hb, g/dL [IQR]	12.7 [12.0–13.7]	13.4 [12.1 – 14.5]	12.7 [11.9 – 14.3]	13.4 [12.2 – 14.2]	0.185	0.357
Platelet, nx10E9/L [IQR]	260 [189–340]	315 [228 – 362]	260[213 – 333]	315 [231 – 368]	0.154	0.099
CRP, mg/L [IQR]	19 [13–19]	22 [7–49]	13 [9–24]	22 [9–51]	0.966	0.382
OPN, ng/ml [IQR]	47 [42–64]	75 [37 – 79]	45 [38–53]	51 [37 – 82]	0.678	0.230
**MPO, ng/mL [IQR]**	**310 [154–444]**	**761 [436–1297]**	**329 [196–465]**	**908 [495–1340]**	**0.003**	** < 0.001**
**MMP-8, ng/mL [IQR]**	**39 **[22–45]	**63 [34–105]**	**40 **[20–55]	**70 [38–109]**	**0.015**	**0.003**
**MMP-9, ng/mL [IQR]**	**1116 [823–1449]**	**2218 [1318–2836]**	**1116 [856–1547]**	**2336 [1370– 2897]**	**0.006**	**0.001**
Resistin, ng/mL [IQR]	15 [10–37]	19 [10–29]	12 [8–26]	20 [11–29]	0.708	0.132
**TIMP-1, ng/mL [IQR]**	243 [237**–** 314]	334 [234**–**430]	**243 [210–320]**	**335 [240–453]**	0.098	**0.026**

Data are presented as median [interquartile range] or absolute (relative) frequencies. Comparisons were drawn by Mann–Whitney test or χ^2^/Fisher’s exact test, as appropriate. P-value* ^§^ refers to comparison between RECIS and iRECIST, respectively

*PD RECIST* progression disease at response evaluation criteria in solid tumors classical or immune-related (iRECIST); *ECOG PS* eastern cooperative oncology group performance status; *SCC* squamous cell carcinoma; *ADK* adenocarcinoma; *WT* wild type; *CT* computerized tomography; *PD* progression disease; *SD* stable disease; *PR* partial response; *CR* complete response

**Table 4:** Variables associated with overall survival (*n* = 79)

	Alive (*n* = 27)	Deceased (*n *= 52)	p-value
Age, years [IQR]	72 [62 – 77]	71 [63 – 77]	0.824
Sex, male (%)	21 (77.8)	38 (73.1)	0.787
Smoke			0.633
never, *n* (%)	1 (3.7)	4 (7.7)	
former, *n* (%)	12 (44.4)	26 (5.0)	
active, *n* (%)	14 (51.9)	36 (45.6)	
**ECOG PS**			**0.012**
**0, *n* (%)**	**14 (51.9)**	**13 (25.0)**	
**1, *n* (%)**	**12 (44.4)**	**25 (48.1)**	
**2, *n* (%)**	**1 (3.7)**	**14 (26.9)**	
Histology			0.327
SCC, *n *(%)	3 (11.1)	13 (25.0)	
ADK, *n *(%)	24 (88.9)	39 (75.0)	
PD-L1 expression			0.819
None, *n* (%)	2 87.49	5 (9.6)	
Mild, *n *(%)	4 (14.8))	10 (19.2)	
High, *n *(%)	21 (77.8)	37 (71.2)	
KRAS mutation* n* (%)	10 (38.5)	18 (40.9)	1.000
Pembrolizumab single agent, *n* (%)	21 (77.8)	35 (67.3)	0.436
**WBC count, nx10E9/L [IQR]**	**9.1 [6.7–10.1]**	**11.5 [8.3–13.5]**	**0.008**
**Neutrophils, nx10E9/L [IQR]**	**6.4 [4.9–8.1]**	**8.1 [5.7–11.2]**	**0.026**
Hb, g/dL [IQR]	13.3 [12.1–14.7]	13.2 [12.0–14.1]	0.487
**Platelet, nx10E9/L [IQR]**	**246 [192–329]**	**331 [254–370]**	**0.012**
CRP, mg/L [IQR]	17 [7–36]	22 [10–50]	0.720
OPN, ng/ml [IQR]	45 [34–55]	52 [39–82]	0.106
**MPO, ng/mL [IQR]**	**390 [273–1032]**	**813 [484–1316]**	**0.020**
MMP-8, ng/mL [IQR]	43 [25–87]	67 [38–106]	0.059
**MMP-9, ng/mL [IQR]**	**1316 [930–2253]**	**2336 [1408–2861]**	**0.019**
Resistin, ng/mL [IQR]	14 [9–28]	19 [11–30]	0.202
**TIMP-1, ng/mL [IQR]**	**243 [200–320]**	**350 [260–453]**	**0.008**

Data are presented as median [interquartile range] or absolute (relative) frequencies. Comparisons were drawn by Mann–Whitney test or χ^2^/Fisher’s exact test, as appropriate. Data are presented as median [interquartile range] or absolute (relative) frequencies

*ECOG PS* eastern cooperative oncology group performance status; *SCC* squamous cell carcinoma; *ADK* adenocarcinoma; *WBC* white blood cell; *Hb* hemoglobin; *CRP* C-reactive protein; *OPN* osteopontin; *MPO* myeloperoxidase; *MMP* metalloproteinase; *TIMP* tissue inhibitor of metalloproteinase

**Fig. 3:**
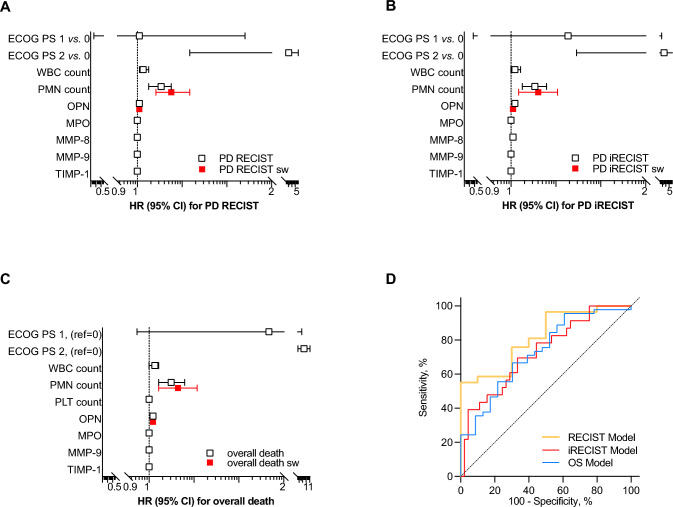
Osteopontin predicts late clinical response to therapies in advanced non-small cell lung cancer. Performance status (ECOG; Eastern Cooperative Oncology Group) White blood cell (WBC) and neutrophil (PMN) counts, matrix metalloproteinase (MMP), myeloperoxidase (MPO), osteopontin (OPN), and tissue inhibitor of matrix metalloproteinase (TIMP) were tested as predictor of and late response defined by progression disease (PD) assessed by both immune-related or not criteria (iRECIST and RECIS, respectively) and presented as hazard ratio (HR) with 95% confidence interval (CI) (**A** to **C**). Models then built were then test at receiver operator characteristic (ROC) curve analysis (**D**)

At Cox regression hazard models, OPN shared with neutrophil count a substantial predictive ability toward both RECIST-PD (HR for OPN 1.01 [1.00 to 1.02]; *p* = 0.017) and iRECIST-PD (HR for OPN 1.01 [1.00 to 1.02]; *p* = -0.005), and overall death as well (HR for OPN 1.02 [1.01 to 1.03]; *p* = 0.001) (Fig. 3 A, B, and C). For all those outcomes, ROC curve analysis confirmed a good discriminating value with AUC of 0.820, 0. 710, and 0. 730, respectively (Fig. 3 D and Supplementary materials). Finally, all the results of Cox regression analyses were internally validated by bootstrap analyses (Supplementary material).

## Discussion

The strong association between OPN and outcome of NSCLC in patients treated with pembrolizumab – alone or in combination with chemotherapy – can be considered the major finding of the present study. This evidence may sound not cutting-edge, as the role of OPN on cancer biology and clinical outcomes has been widely reported in the last decade. A plethora of studies confirmed the predictive role of circulating OPN toward outcome of NSCLC at different stages (Table 5). Even when pooled in a meta-analysis, OPN remained independently associated with more advanced tumor stage at TNM and poor prognosis in terms of disease/progression-free survival and OS [11]. Later studies highlighted a similar predictive value in patients with driver mutation [18] or treated with first-generation immune checkpoint inhibitors [16]. We here moved a step forward by confirming previous results in the setting of pembrolizumab-based regimens. In addition to – and likely because of – a strong independent association with performance status, high serum OPN levels at baseline were independently associated with short-term responses to treatment also, as defined by first/best radiological assessment according to RECIST and iRECIST criteria. Explanations and clinical relevance of these findings need to be framed in the pleiotropic properties of OPN at different stages of cancer biology and beyond. In its secreted form, OPN is indeed being consolidating as stirring regulatory factor or rather the tip of the iceberg of senescence-associated secretory phenotype (SASP) in different classes of disease [19].

**Table 5:** Clinical studies reporting a prognostic role of circulating levels of osteopontin

Author	Year	Patients and treatment regimen	Assay method	Results
Chang YS et al.[64]	2007	130 NSCLC at diagnosis	▪ Serum concentrations ▪ (R&D quantikine kit) ▪ SNPs in OPN gene promoter	▪ Stage of disease was the only independent determinant of serum OPN (p for trend 0.003) ▪ Variation at NT-156 had a significant influence on circulating OPN (p = 0.003)
Mack PC et al. [65]	2008	172 Advanced/metastatic NSCLC Phase III trial of carboplatin/paclitaxel with or without tirapazamine	▪ Serum concentrations ▪ (R&D quantikine kit)	▪ Lower OPN levels determined high rate of response (OR 2.2; p = 0.03) and better PFS (HR = 1.05; p = 0.01) and OS (HR = 1.09, p < 0.001)
Isa S et al. [66]	2009	71 advanced NSCLC phase III trial investigating P + C vs. V + G and D	▪ Serum concentrations ▪ (R&D quantikine kit)	▪ OPN in the lowest quartile showed better PFS (HR: 0.23; p = 0.001) and OS (HR: 0.21; p < 0.001)
Blasber JD et al. [67]	2010	60 pre-surgery early-stage NSCLC	▪ Serum concentrations (ImmunoBiological Laboratories)	▪ OPN significantly decrease after radical surgery ▪ OPN at relapse was elevated from post-surgery nadir
Takenaka M et al. [68]	2013	244 pre-surgery	▪ Serum concentrations ▪ (R&D kit)	▪ 5-year survival after surgery was higher in the lower OPN independently of stage and histotype (HR 0.44 [95%CI 0.225 – 0.77]) ▪ Serum OPN was higher in patients with pleural, microvascular or lymphatic invasion
Ostheimer C et al. [69]	2014	69 Locally advanced inoperable NSCLC candidates to RT	▪ Serial serum concentrations ▪ (IBL Ltd)	▪ Pre-treatment OPN levels were associated with T stage (p = 0.03), M + (p < 0.001 at all time points) and weight loss (p = 0.01) ▪ Stable/Increasing OPN levels during treatment was associated with higher risk of relapse (RR 2.9 [1.3 – 6.6] ▪ High pre-treatment levels of OPN led to reduced OS (p = 0.03) ▪ Rising OPN levels after treatment significantly increased death risk (HR 2.8 [1.3 – 6.2])
Fidler MJ et al. [70]	2018	127 salvage chemotherapy (n = 57) and erlotinib (n = 70 non-EGFR mutated)	▪ MILLIPLEX® MAP ▪ (Millipore Corp.)	▪ OPN was significantly tightly associated with VeriStrat classification ▪ OPN was independent predictor of OS (p = 0.02)
Suwinski R et al.[71]	2019	337 Advanced NSCLC at different stages	▪ Serum concentrations ▪ (Wallac Victor Multi-label Counter)	▪ OPN was significantly associated with 3-year OS (HR 2.09 [1.47 – 2.99]; p > 0.001), but not 5-year OS or locoregional control
Carbone F et al. [72]	2019	74 Advanced pre-treated NSCLC (TNM IIIb or IV) treated with Nivolumab	▪ Serial serum concentrations ▪ (R&D kit)	▪ Baseline OPN was associated with inflammatory status, ECOG-PS (p for trend 0.029) and metastatic burden (p = 0.006) ▪ High OPN levels also predicted worse clinical response (p for trend 0.019) and OS (HR 1.01 [1.00 – 1.01]; p = 0.046)
Xu C et al. [73]	2020	96 Advanced NSCLC Treated with chemotherapy with EP or EC with or without radiotherapy	▪ Serum concentrations ▪ (R&D kit)	▪ After – but not pre- – treatment levels of OPN correlated with treatment response ▪ Increase in after-treatment levels of OPN was associated with higher death risk (HR 4.94 [1.66 – 22.51])

*NSCLC* non-small-cell lung cancer; *SNP* single nucleotide polymorphisms; *OPN* osteopontin; *OR* odds ratio; *HR* hazard ratio; *OS* overall survival; *P* paclitaxel; *C* carboplatin; *V* vinorelbine; *G* gemcitabine; *D* docetaxel; *RT* radiotherapy; *CI* confidence interval; *RT* radiotherapy; VeriStrat VeriStrat test classifies patients as either good or poor based on a matrix assisted laser desorption ionization time-of-flight (MALDI-TOF) mass spectrometry protein signature; EP: etoposide + cisplatin; EC: etoposide + carboplatin

New intriguing hypotheses link senescence, aging and multisystem age-related diseases: from bone metabolism to central nervous, cardiometabolic, and immune systems, till tumor development and progression [20]. For instance, frailty and cognitive decline rise with age among lung cancer patients with a negative – but not standardized yet – impact on OS [21]. The ECOG scale is still of common and valuable use in clinical practice, but there is maybe room for reappraising it [22]. The Cancer and Aging Research Group is therefore validating clinical tools for geriatric assessment and prediction of toxicity [23–26]. Whether panels for senescent secretome may further implement characterization of performance status remains a fascinating but unexplored field of research.

Contrariwise, local aging-related gene signatures are widely known to influence risk stratification and prognosis of lung cancer [27, 28]. They indeed fuel different pathways active on tumor development [29–31], progression [32–35], and resistance to treatment [36]. Detrimental effects of SASP are not limited to malignant cells but involve the whole tumor microenvironment [37, 38] with mutual interactions between endothelial cells [39], fibroblast [40, 41], and immune cells also.

Alongside tumor and physiological aging processes, other comorbidities are known to influence immune system homeostasis. Independently of the trigger, immune senescence targets antigen presentation and then innate immunity: dendritic cells impaired their function, whereas pro-inflammatory monocytes (CD16^+^) prevail on phagocytic ones (CD16^−^) [42]. Tumor-associated macrophages and neutrophils (TAMs and TANs, respectively) locally boost inflammaging and immunosuppression further [38, 43]. Adaptive immunity experiences senescence also. Memory and late-differentiated T-cells accumulate as they lose the expression of co-stimulatory molecules, are less prone to apoptosis, and reduce sensitivity to cytokines and proliferative signals. T cell exhaustion is a late phenotype induced by chronic inflammation and tumor development. It is characterized by loss of effector function, suppressive microenvironment with loss of anti-tumor response function and expression of inhibitory receptors, to which PD-1 belongs [44, 45]. A similar exhausted phenotype may also occur in tissue-resident NK cells [46].

Not limited to lung cancer cells, OPN expression in tumor microenvironment ranges from osteoblasts to fibroblasts, dendritic, lymphoid, and mononuclear cells, but mainly the TAMs [10]. OPN expression in TAMs – but not in cancer cells – has clinical relevance for the outcome of lung cancer [47]. A recent study based on single-cell RNA-seq revealed that OPN expression further differs across different TAM populations, being highly representative of a subset that originates from circulating monocytes [48, 49]. More generally, serum levels of OPN may be expression of whole-body immune senescence [50]. OPN production increases in the subset of senescent PD-1 + T-cells [51] and in age-related diseases such as cardiometabolic ones [52, 53], where co-localize with cell senescent and exhausted T cells [44, 54, 55]. Whether circulating OPN may be finally representative of the abovementioned TAM subset and/or rather expression of systemic senescence is an intriguing but still speculative hypothesis [56].

The rising interest toward the role of senescence and OPN in lung cancer comes up to drug resistance. Both senescence and OPN interact with chemotherapy by promoting each other in experimental studies, but any clinical correlation or relevance has not been proven yet [42]. Rather, such deep immunological implications of senescence and OPN provide a stronger rationale for testing it in the subset of NSCLC candidate to treatments with immune checkpoint inhibitors [57, 58]. All supposed mechanisms of resistance are indeed potentially associated with increased OPN expression [2]. The wide predictive value of OPN here observed – ranging from early to late response – conceptually refers to both innate and acquired resistance mechanisms.

Circulating OPN shares a substantial predictive power with neutrophil count also without any association between them. This should not be surprising as chemotactic properties of OPN are known [59], whereas neutrophil do not release OPN. Rather, circulating neutrophils correlate with TAN [60], which are implicated in carcinogenesis, tumor growth and dissemination [61], and prognostic outcome of immunotherapy as well [62].

To further link our findings with clinical response to therapy, we applied iRECIST criteria that take into account for the early paradoxical responses (i.e., a transient increase of lesion size or the appearance of new small lesions during in the first months of therapy), likely due to the on-site migration of T cell [63]. Therefore, each response other than SD was confirmed with a subsequent radiological assessment at least one month after. Nevertheless, the clinical design of our study leaves to speculation any pathophysiological considerations. The small sample size makes the study unpowered thus limiting result generalization in a broader context. Different demographic, anthropometric, and clinical settings are indeed essential to build a comprehensive and reliable prediction model for testing the real predictive value of OPN. This represents the main limitation of the study. Moreover, wide distribution and pleiotropic activities of OPN intrinsically still limit the identification of – and the extent to which – OPN sources contribute to performance status, and early/late response to pembrolizumab. In addition, OPN is increasingly recognized as poor targetable molecule because of its pleiotropic activity. Rather, we expected more for clinical studies addressing the prognostic role of OPN. OPN is an upstream biomarker directly released by macrophage and meets basic requirements for a good biomarker, being easy to detect and quantify in different biological samples through affordable assays. The biomarker we are looking for should not inform on 5-year OS only, but rather predict who may have a benefit from immune checkpoint inhibitors. We here suggest here that OPN may summarize determinants of poor prognosis in advanced NSCLC: tumor aggressiveness, immunosuppressive microenvironment, and poor performance status. Future studies are called to confirm our preliminary findings in larger clinical cohorts, able to test the value of OPN in adjusted models. We would pay particular attention to the role of visceral adipose tissue as a leading source of OPN. In the era of ‘reverse cardio-oncology’, establishing whether OPN entangles dysfunctional adipose tissue and cancer risk is a relevant question to be solved.

Indeed, they are both influenced by senescence processes, of which OPN is emerging as a pillar. The study of dynamic changes in serum OPN is another—easy to implement—limitation that would add information on treatment response.

In conclusion, we here reported a substantial association of OPN with performance status and short/long term outcomes in patients with advanced NSCLC candidate to pembrolizumab-based regimens. Although preliminary, our data indicate a potential predictive role that would consider local and systemic determinants, basically related to senescence processes.

### Supplementary Information

Below is the link to the electronic supplementary material.

Supplementary file1 (DOC 1877 KB)

## Data Availability

No datasets were generated or analysed during the current study.
