# Supplementary material for: Circulating Osteopontin Predicts Clinical and Radiological Response in First-Line Treatment of Advanced Non-Small Cell Lung Cancer
Source: Lung. 2024 Mar 13;202(2):197–210. doi: 10.1007/s00408-024-00675-5 (PMC11009777; doi:10.1007/s00408-024-00675-5)
Supplement: Supplementary file 1 — Supplementary file1 (DOC 1877 KB) [file 408_2024_675_MOESM1_ESM.doc]

**Table S1. Association of clinical and biochemical biomarkers with PD-L1 expression.**

|  | **PD-L1** | | |  |
| --- | --- | --- | --- | --- |
|  | **<1%** | **1-49%** | **≥50%** | **p-value** |
| Age, years [IQR] | 71 [70 – 74] | 70 [61 – 74] | 71 [62 – 77] | 0.754 |
| Sex, male (%) | 6 (85.7) | 10 (71.4) | 43 (74.1) | 0.764 |
| Smoke |  |  |  | 0.020 |
| never, n (%) | 0 (0.0) | 3 (21.4) | 2 (3.4) |  |
| former, n (%) | 2 (28.6) | 3 (21.4) | 33 (56.9) |  |
| active, n (%) | 5 (71.4) | 8 (57.1) | 23 (39.7) |  |
| ECOG PS |  |  |  | 0.524 |
| 0, n (%) | 3 (42.9) | 4 (28.6) | 20 (34.5) |  |
| 1, n (%) | 2 (28.6) | 9 (64.3) | 26 (44.8) |  |
| 2, n (%) | 2 (28.6) | 1 (7.1) | 12 (20.7) |  |
| Histology |  |  |  | 0.026 |
| SCC, n (%) | 0 (0.0) | 0 (0.0) | 0 (27.6) |  |
| ADK, n (%) | 7 (100.0) | 14 (100.0) | 42 (72.4) |  |
| KRAS mutation, n (%) | 4 (57.1) | 4 (30.8) | 20 (40.0) | 0.517 |
| WBC count, nx10E9/L [IQR] | 8.6 [8.0 – 16.0] | 10.2 [6.7 – 14.3] | 9.9 [8.3 – 13.0] | 0.994 |
| Neutrophils, nx10E9/L [IQR] | 6.5 [5.4 – 11.1] | 7.6 [4.9 – 10.7] | 7.5 [5.4 – 9.9] | 0.928 |
| Hb, g/dL [IQR] | 12.2 [11.6 – 14.1] | 13.5 [12.4 – 14.9] | 13.4 [12.4 – 14.9] | 0.580 |
| Platelet, nx10E9/L [IQR] | 232 [189 – 343] | 316 [187 – 396] | 311 [237 – 353] | 0.752 |
| CRP mg/L | 3 [3 – 3] | 9 [3 – 9] | 22 13 – 51] | 0.222 |
| OPN, ng/mL [IQR] | 48 [26 – 88] | 50 [41 – 85] | 49 [37 – 74] | 0.702 |
| MPO, ng/mL [IQR] | 697 [382 – 1820] | 512 [265 – 2312] | 727 [328 – 1198] | 0.772 |
| MMP-8, ng/mL [IQR] | 45 [40 – 61] | 70 [28 – 136] | 56 [30 – 97] | 0.578 |
| MMP-9, ng/mL [IQR] | 1377 [1074 – 2243] | 2634 [1497 – 2910] | 1741 [1138 – 2749] | 0.341 |
| Resistin, ng/mL [IQR] | 12 [10 – 22] | 18 [9 – 35] | 19 [10 – 29] | 0.643 |
| TIMP-1, ng/mL [IQR] | 275 [241 – 334] | 315 [223 – 504] | 314 216 – 430] | 0.733 |

Data are presented as median [interquartile range]. Comparisons were drawn by Chi square or Kruskal-Wallis test, as appropriate.

ECOG PS: eastern cooperative oncology group performance status; SCC: squamous cell carcinoma; ADK: adenocarcinoma; WBC: white blood cell; Hb: hemoglobin; eGFR: estimated glomerular filtration rate; CRP: C-reactive protein; OPN: osteopontin; MPO: myeloperoxidase; MMP: metalloproteinase; TIMP: tissue inhibitor of metalloproteinase.


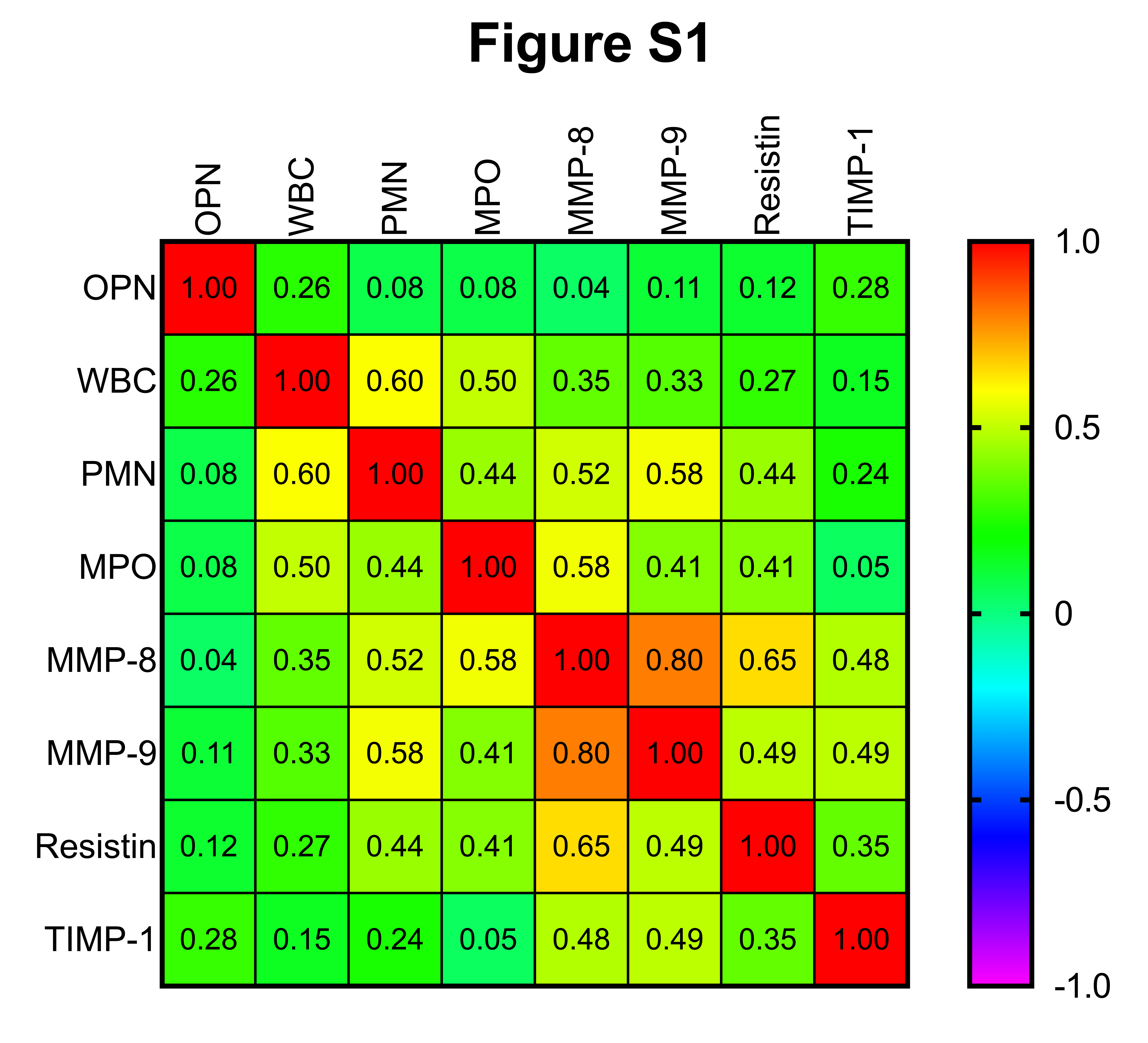


# Figure S1. Correlation matrix reporting the correlation between inflammatory biomarkers.

**Table S2. Association of clinical and biochemical biomarkers with disease characteristics.**

|  | **ECOG PS** | | |  |
| --- | --- | --- | --- | --- |
|  | **0** | **1** | **2** | **p-value** |
| Age, years [IQR] | 69 [61 – 74] | 72 [65 – 79] | 71 [67 – 77] | 0.239 |
| Hb, g/dL [IQR] | 13.2 [11.9 – 14.6] | 13.4 [12.3 – 14.4] | 12.9 [12.2 – 14.1] | 0.818 |
| Platelet, nx10E9/L [IQR] | 271 [214 – 343] | 316 [244 – 357] | 314 [248 – 411] | 0.455 |
| CRP mg/L | 18 [4 – 51] | 19 [13 – 41] | 22 [11 – 62] | 0.912 |
| MPO, ng/mL [IQR] | 714 [330 – 1198] | 635 [305 – 1259] | 1012 [441 – 1354] | 0.655 |
| MMP-8, ng/mL [IQR] | 48 [27 – 82] | 64 [36 – 110] | 83 [36 – 109] | 0.332 |
| Resistin, ng/mL [IQR] | 12 [9 – 29] | 20 [13 – 31] | 19 [9 – 26] | 0.256 |
| TIMP-1, ng/mL [IQR] | 261 [201 – 365] | 314 [237 – 460] | 358 [329 – 430] | 0.094 |

Data are presented as median [interquartile range]. Comparisons were drawn by Kruskal-Wallis test. No differences in neither ECOG PS nor inflammatory biomarkers across sex, smoker habit, histology, PD-L1 expression, KRAS mutation or therapy regimen were found.

Hb: hemoglobin; CRP: C-reactive protein; MPO: myeloperoxidase; MMP: metalloproteinase; TIMP: tissue inhibitor of metalloproteinase.

**Table S3. Variables associated with at the 1^st^ CT response (n=78; excluded the only case of CR).**

|  | **PR**  **(n=11)** | **SD**  **(n=37)** | **PD**  **(n=16)** | **Early death**  **(n=14)** | **p-value** |
| --- | --- | --- | --- | --- | --- |
| Age, years [IQR] | 76 [65 – 80] | 72 [63 – 76] | 68 [59 – 73] | 70 [61 – 73] | 0.105 |
| Hb, g/dL [IQR] | 13.8 [12.8 – 14.4] | 13.0 [11.9 – 14.6] | 13.2 [12.1 – 13.9] | 13.5 [12.2 – 14.3] | 0.721 |
| Platelet, nx10E9/L [IQR] | 244 [214 – 293] | 271 [212 – 342] | 335 [286 – 410] | 337 [284 – 337] | 0.060 |
| CRP mg/L | 11 [5 – 14] | 24 [15 – 49] | 37 [5 – 56] | 22 [14 – 52] | 0.256 |
| MPO, ng/mL [IQR] | 730 [237 – 1560] | 505 [311 – 1333] | 620 [441 – 1222] | 809 [631 – 1079] | 0.908 |
| MMP-8, ng/mL [IQR] | 61 [40 – 124] | 43 [29 – 96] | 61 [37 – 98] | 86 [55 – 117] | 0.217 |
| Resistin, ng/mL [IQR] | 21 [7 – 36] | 17 [10 – 28] | 14 [9 – 25] | 18 [17 – 41] | 0.437 |
| TIMP-1, ng/mL [IQR] | 335 [204 – 456] | 284 [196 – 387] | 334 [295 – 477] | 395 [241 – 576] | 0.093 |

Data are presented as median [interquartile range]. Comparisons were drawn by Kruskal-Wallis test. No differences in neither ECOG PS nor inflammatory biomarkers across sex, smoker habit, histology, PD-L1 expression, KRAS mutation or therapy regimen were found.

Hb: hemoglobin; CRP: C-reactive protein; MPO: myeloperoxidase; MMP: metalloproteinase; TIMP: tissue inhibitor of metalloproteinase.

**Table S4. Variables associated with at the best response (n=64; excluded the only case of CR).**

|  | **PD**  **(n=15)** | **SD**  **(n=28)** | **PR**  **(n=21)** | **p-value** |
| --- | --- | --- | --- | --- |
| Age, years [IQR] | 70 [59 – 73] | 71 [65 – 75] | 76 [65 – 80] | 0.149 |
| Hb, g/dL [IQR] | 13.1 [12.0 – 14.0] | 13.0 [11.7 – 14.2] | 13.7 [12.5 – 15.1] | 0.358 |
| Platelet, nx10E9/L [IQR] | 336 [325 – 426] | 284 [208 – 350] | 245 [218 – 285] | 0.069 |
| CRP, mg/L [IQR] | 42 [4 – 60] | 19 [10 – 47] | 14 [8 – 35] | 0.778 |
| MPO, ng/mL [IQR] | 512 [393 – 1233] | 1082 [336 – 1495] | 441 [237 – 966] | 0.203 |
| MMP-8, ng/mL [IQR] | 70 [34 – 107] | 52 [32 – 97] | 42 [29 – 86] | 0.703 |
| Resistin, ng/mL [IQR] | 12 [9 – 23] | 19 [10 – 30] | 19 [8 – 29] | 0.455 |
| TIMP-1, ng/mL [IQR] | 334 [308 – 491] | 290 [209 – 381] | 299 [191 – 399] | 0.126 |

Data are presented as median [interquartile range]. Comparisons were drawn by Kruskal-Wallis test. No differences in neither ECOG PS nor inflammatory biomarkers across sex, smoker habit, histology, PD-L1 expression, KRAS mutation or therapy regimen were found.

Hb: hemoglobin; CRP: C-reactive protein; MPO: myeloperoxidase; MMP: metalloproteinase; TIMP: tissue inhibitor of metalloproteinase.

**Statistical considerations about sample size calculation, performance (calibration, discrimination, and internal validation)**

According with the guidelines for sample size calculation (<https://www.bmj.com/content/368/bmj.m441>), for a binary outcomes:

- 1. In the case of models with binary outcome, a 95% confidence interval for the overall outcome proportion of 0.5 is recommended. This requires at least **358** subjects (Equation 1, Figure 1 of (Riley et al, 2020). More specifically, the outcome proportion for progression disease at 6 months (corresponding to our median follow-up time) has been previously estimated in about 37% for treatment with pembrolizumab, both alone or in combination with chemotherapy (Reck M, et al. N Engl J Med. 2016; Gandhi L, et al. N Engl J Med. 2018).
  2. As models with binary outcome, a Mean Absolute Precision Error (MAPE) < 0.05 is recommended. Given an overall outcome proportion of 0.5, and having 4 predictors, at least **114** subjects are required in order to obtain the desired MAPE (Equation 2, Figure 2 of (Riley et al, 2020), with Φ=0.06, P=3, MAPE = 0.05).
  3. In the case of models with binary outcome, an expected uniform shrinkage factor <10% is recommended. Having 3 candidate predictors, at least **170** subjects are required, with P=3, S=0.9, R^2^_cs_=0.1.
  4. The model’s proportion of overall variance explained (R^2^_cs_) requires to be pre-specified in order to compute the expected uniform shrinkage factor (see point c). According to Figure 3 and 4 of (Riley et al, 2020), we select R^2^_cs_=0.1.

**Model discrimination** performances are already presented via the ROC curve analysis (Table S4). Sensitivity and specificity of ROC curves are presented below:

|  | AUC | 95% CI | p-value |
| --- | --- | --- | --- |
| RECIST | 0.819 | 0.707 – 0.902 | <0.001 |
| iRECIST | 0.706 | 0.583 – 0.810 | 0.005 |
| OS | 0.730 | 0.608 – 0.930 | <0.001 |

**Bootstrap resampling performance**.

Based on 1000 bootstrap replicates, we obtained the following estimate of the HR (average of the 1000 HRs from the 1000 bootstrap samples) and of their 95% confidence interval. When the HR estimated from the original dataset are considered, we note that they fall within the new bootstrap confidence intervals.

Bootstrap for RECIST

Neutrophils HR 1.20 [1.12 – 1.31]; p=0.001

OPN HR 1.00 [1.00 – 1.00]; p=0.041

Bootstrap for iRECIST

Neutrophils HR 1.15 [1.03 – 1.29]; p=0.048

OPN HR 1.00 [1.00 – 1.00]; p=0.017

TIMP-1 HR 1.00 [1.00 – 1.00]; p=0.036

Bootstrap for OS

Neutrophil: HR 1.16 [1.06 – 1.29] p=0.002

OPN: 1.02 [1.01 – 1.03] p=0.002
